# Aquaporins: Gatekeepers of Fluid Dynamics in Traumatic Brain Injury

**DOI:** 10.3390/ijms25126553

**Published:** 2024-06-14

**Authors:** Wojciech Czyżewski, Jakub Litak, Jan Sobstyl, Tomasz Mandat, Kamil Torres, Grzegorz Staśkiewicz

**Affiliations:** 1 Department of Neurosurgery, Maria Sklodowska-Curie National Research Institute of Oncology, ul. W.K. Roentgena 5, 02-781 Warsaw, Poland; tomaszmandat@yahoo.com; 2 Department of Didactics and Medical Simulation, Medical University of Lublin, 20-954 Lublin, Poland; 3 Department of Clinical Immunology, Medical University of Lublin, 20-954 Lublin, Poland; jakub.litak@gmail.com; 4 Department of Interventional Radiology and Neuroradiology, Medical University of Lublin, 20-954 Lublin, Poland; jan.sobstyl@gmail.com; 5 Department of Plastic, Reconstructive Surgery with Microsurgery, Medical University of Lublin, 20-954 Lublin, Poland; kamiltorres@wp.pl; 6 Department of Human, Clinical and Radiological Anatomy, Medical University, 20-954 Lublin, Poland; grzegorz.staskiewicz@gmail.com

**Keywords:** aquaporin, AQP1, AQP4, AQP9, TBI, brain edema

## Abstract

Aquaporins (AQPs), particularly AQP4, play a crucial role in regulating fluid dynamics in the brain, impacting the development and resolution of edema following traumatic brain injury (TBI). This review examines the alterations in AQP expression and localization post-injury, exploring their effects on brain edema and overall injury outcomes. We discuss the underlying molecular mechanisms regulating AQP expression, highlighting potential therapeutic strategies to modulate AQP function. These insights provide a comprehensive understanding of AQPs in TBI and suggest novel approaches for improving clinical outcomes through targeted interventions.

## 1. Introduction

Traumatic brain injury (TBI) represents a significant public health challenge, affecting millions globally and serving as a leading cause of death and disability [1,2,3]. It results from an external mechanical force, such as a blow or jolt to the head, which can lead to a wide range of functional short- or long-term changes affecting thinking, sensation, language, or emotion [4]. These injuries can also lead to profound physical and psychological effects and are a major cause of morbidity and prolonged hospitalization [4,5,6].

TBI is a complex injury with a broad spectrum of symptoms and disabilities. The impact on a person and their family can be devastating [7]. According to the World Health Organization, TBI will surpass many diseases as the major cause of death and disability by the year 2030 [8,9]. This underscores the urgent need for enhanced diagnostic techniques, better treatment protocols, and more effective rehabilitation strategies to manage the consequences of TBI. The financial burden on healthcare systems and families is enormous, as prolonged treatment and loss of productivity impact society significantly [10,11].

Following the initial injury, the pathology of traumatic brain injury (TBI) often progresses, leading to secondary brain damage that can be more detrimental than the initial injury [12,13]. One of the most critical secondary effects is cerebral edema, which is a major determinant of morbidity and mortality following TBI [13,14]. This swelling results from the influx of fluids into brain tissues, leading to increased intracranial pressure (ICP), which can compress and damage delicate brain structures [15]. Managing cerebral edema is crucial in the acute phase of TBI treatment to preserve brain function and improve outcomes [16].

Aquaporins (AQPs) are a family of water-channel proteins that facilitate the transport of water and other small molecules across cell membranes [17]. In the brain, several aquaporins have been identified, but AQP4 is the most abundant and plays a critical role in maintaining water homeostasis. AQP4 is predominantly expressed in the foot processes of astrocytes, which are intimately associated with the brain’s blood vessels [18,19]. This strategic localization suggests a significant role in forming the blood–brain barrier (BBB) and in water movement within the brain, making it a critical player in the development and resolution of cerebral edema [20].

Understanding the dynamic regulation of AQP4 and other aquaporins in the brain is essential for deciphering their roles in both normal physiology and pathological conditions like TBI. The discovery of aquaporins has enabled a new look at the pathophysiology of water homeostasis disorders in the brain. Many recent studies have demonstrated the impact of AQP1, AQP4, and AQP9 on water balance and brain edema pathology, both in the cytotoxic and vasogenic mechanisms [21]. Currently, treatment for brain edema is limited to reducing intracranial pressure; however, there are no methods to combat it at the molecular level. Research into AQPs may offer new therapeutic targets to modulate water transport in efforts to control edema and mitigate secondary injuries in TBI patients, providing new avenues for clinical interventions. A full understanding of the function of aquaporins in the pathomechanism of brain edema may allow for the identification of potential therapeutic agents to inhibit this pathophysiological process. This review aims to explore the implications of aquaporin function in traumatic brain injury, emphasizing the potential of AQPs as therapeutic targets to improve outcomes for those affected by this challenging condition.

## 2. Aquaporins in the CNS

Aquaporins mediate osmotically and hydrostatically driven water transport between four aqueous spaces in the nervous system: intracellular fluid, interstitial fluid, cerebrospinal fluid, and blood [22,23]. Until now, thirteen subtypes of the aquaporin family have been detected in humans (AQP0-AQP12). To date, most types of aquaporins have been identified in various areas of the nervous system: AQP1 [24], AQP2 [25], AQP3 [26], AQP4 [27], AQP5 [28], AQP6 [29], AQP7 [30], AQP8 [31], AQP9 [32], and AQP11 [33,34]. Moreover, AQPs 0, 6, and 10 have been identified at the transcript level in human brains—a finding that has not been reported before [35]. Among these, aquaporins 1, 4, and 9 are well described in pathologies of the central nervous system and are involved in processes such as brain edema, tumor angiogenesis, autoimmune diseases, and the formation of glial scars [36].

While other aquaporins have been identified in the CNS, their roles in pathological processes remain largely unexplored. The function of AQP4 is more extensively documented, particularly in the brain, spinal cord, and optic nerve [37]. In the brain, it is typically located on the surfaces of subarachnoid astrocyte processes, forming a glial-limiting membrane (serving as a junction at the border of the central nervous system and cerebrospinal fluid) around the perivascular end-feet of astrocytes (forming the interface between the central nervous system and blood), and on the basolateral membranes of ependymal cells and subependymal astrocyte processes (creating an interface between the central nervous system and cerebrospinal fluid) [38]. The density of AQP4 is predominantly localized to the region of the astrocyte proximal to the vascular interface, illustrating a polarized expression pattern of AQP4 [18]. The pattern of this aquaporin’s occurrence at the borders between brain tissues and cerebral fluid spaces suggests the function of this aquaporin in cellular bidirectional water transport [39,40].

Although these aquaporins are mostly found in the blood–brain barrier, they are also found in brain regions where it does not exist, such as in the circumventricular organs [41,42], which include the posterior lobe of the pituitary gland, the vascular organ of the terminal lamina, the subfornical organ, the subcommissural organ, area postrema, the pineal gland, and the medial eminence of the hypothalamus [43]. 

AQP1 is expressed throughout the mammalian nervous system, showing a diverse range of localizations both in the central and peripheral nervous systems. Within the brain, AQP1 is found in the choroid plexus epithelia of humans and mice—a critical area for the production and regulation of cerebrospinal fluid [44]. Additionally, it is present in mouse olfactory ensheathing glia—cells that play a crucial role in the sense of smell and neural regeneration [45]—and has been detected in human astrocytes [46]. Using the uDISCO tissue clearing method and Western blot analysis, Q. Li and colleagues in 2020 attempted to determine the localization of AQP1 in the vessels of the brain and spinal cord. The study results indicate a widespread presence of these aquaporins in the smooth muscle of the middle meningeal artery, arterioles, and veins, as well as in the endothelial cells of arterioles and venules of the soft meninges and arachnoid [47]. The study also found a lack of AQP1 in interstitial vessels except for a small portion of penetrating cortical arterioles. In the peripheral nervous system, AQP1 expression is observed in a subset of primary sensory neurons located in the dorsal root and nodose ganglia of mice [48]. Furthermore, AQP1 is present in enteric neurons of both mice and rats, indicating its role in the gastrointestinal nervous system, where it likely contributes to fluid transport and neuronal function [49]. It was also described in the human trigeminal system [50]. AQP1 along with AQP4 plays a significant role in the homeostasis of cerebrospinal and interstitial brain fluids. Their location implies a classic, albeit very simplified, scheme of their function in which AQP1 participates in the production of cerebrospinal fluid and AQP4 in the absorption and exchange of cerebrospinal and interstitial fluid [44] (Trillo-Contreras et al, 2019). In reality, the production, exchange, and absorption of fluids within the brain are much more complicated. Currently, the most popular hypothesis is the “Bulat-Klarica-Oreskovic theory,” according to which the production of cerebrospinal fluid is not limited to one organ but is performed through constant fluid exchange between the capillaries of the central nervous system’s blood vessels and the brain’s interstitial fluid [51]. This theory was subsequently supported by studies conducted by Nakada and his research team, who analyzed the passage of water from the brain to the cerebrospinal fluid in genetically deprived AQP1 and AQP4 mice as well as wild-type mice using magnetic resonance diffusion. Their results indicated that the influx of water into the cerebrospinal fluid is regulated with the help of AQP4, which is responsible for the water homeostasis of the perivascular space, and not AQP1 located in the choroid plexus [23,52]. 

AQP9 has been discovered in astrocytes [53], catecholaminergic neurons [32], tanycytes [54], midbrain dopaminergic neurons, hypothalamic neurons [53], and the area of the ventral tegmental area of the midbrain [55,56]. 

The distribution of other AQPs within the CNS was described only sporadically. AQP2 is distributed in the ependymal cell layer, subcortical white matter, and the hippocampus [57]. In the peripheral nervous system, AQP2 is located in structures such as the rat extra-temporal facial nerve, vomeronasal sensory neurons, trigeminal ganglion neurons, and small-diameter dorsal root ganglia neurons, where it may play a role in pain transmission and responses to neuropathic nerve injury [58,59]. Additionally, AQP2 expression has been detected in glioma cells, astrocytes, and microglial cell lines in rats following TBI [25]. AQP3 is distributed in the choroid plexus and was detected in rat astrocytes and neurons [57,60,61]. 

The role of AQP5 in the brain is not yet fully understood, although it is known to be expressed in the astrocytes and ependymal cells of rodents [54]. AQP6, an anion-selective channel, is not commonly expressed in the CNS under normal conditions, suggesting a specialized, possibly regulatory role in ion transport in specific CNS cells during stress or disease [35]. During brain development, AQP7 plays a significant role, particularly in structures related to cerebrospinal fluid (CSF) production and brain hydration. Immunoreactivity studies have shown that AQP7 is predominantly located in the choroid plexus throughout brain development. Additionally, the presence of AQP7 increases in other areas, such as the ependyma, pia mater, and blood vessels from the perinatal to the postnatal stages. Cells within various layers of the cerebral cortex also begin to exhibit slight AQP7 positivity during postnatal development, indicating its broader involvement in neural tissue hydration and possibly neuronal function [30]. AQP7 is also detected in the glial cells and might be involved in glycerol transport associated with energy storage and metabolism [62,63]. AQP8, expressed in some neurons and astrocytes, is thought to contribute to ammonia transport and has been found to be overexpressed in glioma cells [64].

AQP10, although primarily an intestinal aquaporin, has ambiguous roles in the CNS and requires further investigation to define its presence and function. AQP11 is found in ependymal cells and is possibly involved in intracellular water transport mechanisms critical for cellular homeostasis and protection against osmotic stress. Additionally, AQP11 RNA exhibits distinct localization, being found in the dendrites of Purkinje cells, neurons in the CA1 and CA2 regions of the hippocampus, and neurons in the cerebral cortex [65]. To date, the pancreas-specific aquaporin, AQP12, has not been identified in the brain.

## 3. Role of AQPs in TBI

After an initial traumatic brain injury (TBI), a series of complex molecular reactions results in additional damage via inflammation, altered energy metabolism in the brain, or hypoxia, termed secondary damage. This leads to both structural and functional impacts, including the development of brain edema, glial scars, and neuronal loss. These interconnected processes are still not completely comprehended. It is known that AQP4 channels facilitate water entry into CNS tissues, yet the precise origin of this water—whether from the bloodstream, CSF, or perivascular spaces—is still contested [66].

Edema presents a critical issue in the CNS due to the constricted expansion capacity within the skull and spine. In recent years, edema has been categorized into three distinct types: cytotoxic, ionic, and vasogenic, each reflecting different pathological changes in the brain [67]. Cytotoxic edema is marked by the accumulation of water inside cells without damage to the BBB, typically resulting from a decrease in oxygen tension, and is characterized by the swelling of astrocytes and the appearance of beading on neuronal dendrites [68]. Ionic edema, often occurring alongside cytotoxic edema, involves the entry of water and sodium ions into the brain prior to the dysfunction of tight junctions [69]. Conversely, vasogenic edema is triggered by BBB breakdown, allowing plasma proteins and water to seep into brain tissue [70].

Aquaporins in TBI influence both cytotoxic and vasogenic edema [22]. Among the different aquaporins expressed in the brain, AQP4 is the most abundant and has been most closely linked with the pathophysiology of TBI [71]. Water regulation, astrocyte swelling, and blood–brain barrier integrity are key processes influenced by AQP4 in the brain. AQP4 facilitates the rapid movement of water following injury-induced ionic fluxes and osmotic gradients, significantly influencing the development and resolution of cerebral edema [72]. Additionally, AQP4’s modulation affects astrocyte swelling, a critical factor in cytotoxic edema, where cells swell due to the intracellular accumulation of water. Alterations in AQP4 expression and localization can also affect the permeability of the BBB, influencing vasogenic edema, where fluid leaks into the extracellular space of the brain, further complicating the overall impact of brain injuries [73]. 

In cytotoxic edema, water molecules flow into the central nervous system through AQP4 channels located in the perivascular end-feet of astrocytes [74]. In vasogenic edema, water molecules are removed with the help of AQP4 in various ways: directly to the bloodstream through the end-feet, through astrocytic interstitial processes and meningeal cells to the subdural cerebrospinal fluid space, and through subependymal astrocytic processes and ependymal epithelium to the brain ventricular system, also known as the glymphatic system [14]. Research indicates that CSF is the primary contributor to water in AQP4-dependent edema, rather than blood. Therefore, it could be hypothesized that temporarily targeting the choroid plexus to manipulate aquaporins or ion pumps could decrease CSF production, offering a potential method to mitigate edema development [75]. In a study conducted by Braun et al., a post-mortem analysis of the human frontal cortex showed varied laminar expression of AQP4 following blast exposure. Corresponding changes in AQP4 and delayed glymphatic dysfunction were also noted in a mouse model of blast mTBI 28 days post-injury. Veterans with blast mTBI exhibited increased MRI-visible perivascular spaces in the frontal cortex, suggesting glymphatic impairment [76]. 

Determining the contribution of AQP4 in the formation and elimination of edema thus presents a challenge and may be related to the spatiotemporal pattern of their expression. Previous TBI studies show a decrease in AQP4 expression within 48 h after injury (potentially coexisting with vasogenic edema), often at sites of blood–brain barrier disruption [77]. It has also been proven that an increase in receptors for AQP4 in human astrocytomas corresponds with the detection of brain edema in magnetic resonance imaging [78]. Additionally, AQP4 deletion reduces cytotoxic edema caused primarily by water intoxication and cerebral ischemia [79]. In studies conducted by Sun MC et al., an increase in expression was observed in the lobe affected by the injury, there was a decrease in expression in the adjacent part of the brain where edema was most pronounced, and there were no changes in the lobe distant from the site of injury [80].

Huang Y. et al. used a spinal cord contusion (SCC) model performed on lab rodents to examine the role of AQP4 and its interaction with cytochrome coxidase (COX5A), which affects energy metabolism. According to their results, increased AQP4 expression helps manage vasogenic edema by promoting water reabsorption but later contributes to cytotoxic edema. Techniques like RNA interference of AQP4 (AQP4-RNAi) demonstrate that reducing AQP4 helps decrease cytotoxic edema and improve motor functions, potentially due to COX5A upregulation, though the exact mechanism remains unclear [81]. Zhang Y. et al. investigated the effects of a long noncoding RNA, Malat1, on brain swelling after traumatic brain injury (TBI). They observed that Malat1 levels were reduced in brain injury models, which was associated with increased brain edema and astrocyte swelling. This decrease in Malat1 occurred alongside rises in interleukin-6 (IL-6), nuclear factor-kappa B (NF-κB), and aquaporin 4 (AQP4), all linked to inflammation and edema. Significantly, overexpressing Malat1 led to a substantial decrease in brain edema and lowered levels of IL-6, NF-κB, and AQP4, indicating that Malat1 has a protective role by influencing these factors. Consequently, lowering AQP4 expression through Malat1 might reduce water influx into astrocytes, thus reducing the swelling tied to cytotoxic edema [82]. 

The involvement of AQP4 in the removal of brain extracellular interstitial fluid in vasogenic edema was investigated by Papadopoulos et al. When comparing the modified mice with wild-type mice, it appears that the deletion of the aquaporin gene is associated with a disrupted elimination of water molecules. Further observations and comparisons led to the conclusion that in vasogenic edema, water molecules flow into the brain parenchyma independently of AQP4, while their elimination requires its involvement [83]. These observations propose that the specific conditions of the disease environment influence the overall role of AQP4 in the development of cerebral edema. Jeon H. et al. proved that reactive oxygen species (ROS) generated during intracerebral hemorrhage reduce AQP4 expression on astrocytes, which are crucial for maintaining BBB integrity. This reduction disrupts the BBB’s structure and leads to early perihematomal edema. Treating ICH mice with an ROS scavenger significantly decreased PHE by restoring AQP4’s water-clearing function. Additionally, experiments with AQP4-deficient mice and an AQP4 enhancer demonstrated that AQP4 is essential for preventing PHE by maintaining BBB structure [84]. The impact of AQP4 on cerebral edema is underlined by the effect of AQP4 inhibition using AER-270 or AER-271, which resulted in enhanced neurological outcomes and reduced swelling in lab animal models of central nervous system injury [85]. 

AQP1 is found in cortical neurons and is believed to play a role in post-trauma neuronal swelling and the development of cytotoxic brain edema in the ipsilateral hemisphere following experimental TBI, with regulation involving V1a receptors [86]. To evaluate the role of AQP1 in CNS homeostasis post-TBI, Tran et al. found that cerebral edema increased at 4 and 24 h post-TBI. Dexamethasone reduced edema under nonacidotic conditions but worsened it under acidotic conditions, while blocking AQP1 channels with HgCl2 alleviated the effects. AQP1 expression was low in uninjured animals but higher in TBI-affected brains [87]. Based on studies on laboratory animals, Bo Qiu et al. draw conclusions that AQP-1 may participate in edema formation and delayed cell death in the hippocampus following TBI [88].

AQP9 also plays a role in edema prevention and mitigating lactic acidosis under ischemic conditions in human retinal pigment epithelium (RPE) cells [89]. Following an injury, AQP9 upregulation in astrocytes causes morphological changes and increased astrocyte reactivity, potentially resulting in a beneficial effect by decreasing lesion size and aiding in edema resolution in TBI [90].

Although recent research lacks studies investigating AQP5 in trauma settings, it can be hypothesized that AQP5 plays a role in edema formation or resolution based on analogous studies. AQP5 expression is associated with the development and intensity of peritumoral brain edema in meningioma patients, with higher AQP5 levels significantly correlated with increased edema severity. The presence of the A(−1364)C genotype in AQP5 further correlates with greater edema intensity, suggesting AQP5’s involvement in the formation of cerebral edema [28,91]. 

AQP11 is found on brain endothelial cells and contributes to the induction of perihematomal edema (PHE), an effect linked to miR-27a-3p. miR-27a-3p downregulation contributes to brain edema, blood–brain barrier disruption, neuron loss, and neurological deficits following ICH. Exogenous miR-27a-3p may protect against post-ICH complications by targeting AQP11 in the capillary endothelial cells of the brain [92].

## 4. Mechanisms Underlying Aquaporin Dysregulation in TBI

**Figure 1:**
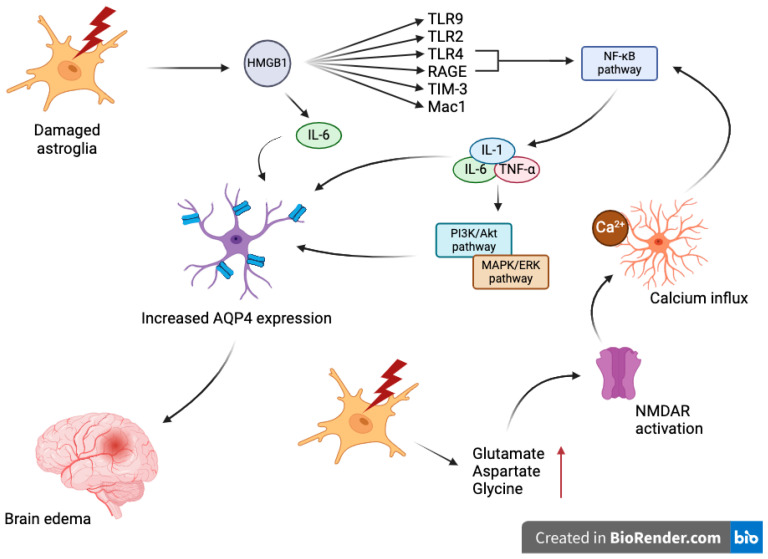
Molecular mechanisms of AQP4’s dysregulation.

The molecular mechanisms regulating AQP4 expression in response to TBI involve several signaling pathways and transcription factors (Figure 1). After a TBI event, microglial cells become highly activated, initiating a cascade of inflammatory responses that can exacerbate the injury. One of the critical molecules released during this process is high-mobility group box 1 (HMGB1), a part of DAMP’s family, which escapes from the nucleus of damaged neurons into the extracellular space. HMGB1 is a potent mediator of inflammation, and upon release, it binds to a plethora of receptors, including Toll-like receptor 4 (TLR4), TLR9, TLR2, T-cell immunoglobulin and mucin domain (TIM-3), microglial macrophage antigen complex 1 (Mac1) and the Receptor for Advanced Glycation Endproducts (RAGE), among many others [93]. During neuroinflammatory states, HMGB1 is actively secreted by neurons and glial cells following inflammasome activation, subsequently activating at least two pattern recognition receptors (PRRs), specifically TLR4 and RAGE, on target cells. This binding triggers the activation of the nuclear factor kappa-light-chain-enhancer of activated B cells (NF-κB) pathway, a pivotal molecular route that controls the transcription of DNA, pro-inflammatory cytokine production, and cell survival [94]. Neuronal HMGB1 also stimulates the release of interleukin-6 (IL-6), which subsequently increases the expression of the water channel protein AQP4 in astrocytes [95,96]. This increase in AQP4 is associated with the management of cerebral edema but can contribute to its pathology under excessive conditions. The anti-HMGB1 monoclonal antibody significantly reduced brain edema caused by fluid percussion by blocking HMGB1 translocation, preserving the integrity of the blood–brain barrier (BBB), reducing the expression of inflammatory molecules, and enhancing motor function [97]. In other in vitro studies, tanshinone IIA (TSO IIA) notably lessened swelling in astrocytes following glucose and oxygen deprivation and reoxygenation (OGD/R) damage, mimicking ischemia–reperfusion-like injury in vitro by inhibiting the activation of the HMGB1/RAGE/NF-κB/IL-6 inflammatory pathway, which in turn lowered the presence of AQP4 in the plasma membrane [98]. Additionally, after TBI, there is a rapid, transient increase in the levels of neurotransmitters like glutamate, aspartate or glycine in the extracellular space, which can lead to excitotoxicity [99]. The increased glutamate activates NMDA receptors on astrocytes, triggering intracellular calcium influx [100]. Elevated intracellular calcium is a critical second messenger in cellular signaling, affecting various pathways regulating gene expression [101] that also affects above-mentioned NF-kB pathway [102,103]. The activation of NF-κB is followed by a surge in the production of pro-inflammatory cytokines such as interleukin-1 (IL-1), interleukin-6 (IL-6), and tumor necrosis factor-alpha (TNF-α) [96]. Once activated, NF-kB translocases to the nucleus, where it can bind to specific sequences in the promoter regions of the AQP4 gene, upregulating its expression [104]. This upregulation is thought to be a compensatory response aimed at removing excess water from the brain parenchyma, although excessive AQP4 expression can exacerbate edema [105]. The aforementioned cytokines activate multiple signaling cascades, including the MAPK and PI3K/Akt pathways, which further modulate the transcription and translation of AQP4 [106,107]. Additionally, the MAPK/ERK signaling pathway, critical in cellular response to stress, is activated, potentially leading to the phosphorylation of transcription factors that increase AQP4 expression, influencing cell survival, proliferation, and inflammatory responses [107,108,109].

In neurons, the activation of transcription factor FoxO3a, which is part of the FoxO family of forkhead transcription factors, can either protect against excitotoxic insults or initiate neuronal death. When protein kinase B (Akt) activates, it phosphorylates Foxo3a at the Ser256 residue, blocking its movement into the nucleus and thus inhibiting its transcriptional activation [110]. Mutating the phosphorylation sites on Foxo3a leads to an increase in its nuclear translocation and transcriptional activity [111]. The activity of FoxO3a is modulated by signaling pathways such as PI3K/Akt and microRNAs (miRNAs) [112]. TBI activates Foxo3a transcriptionally, which then promotes the expression of AQP4 at the injury site 24 h post-TBI. Reducing Foxo3a levels in mice prevents the increase in AQP4 in the brain, diminishes cerebral edema, and enhances neurological outcomes following TBI [113].

Protein kinase C (PKC) downregulates AQP4 expression in astrocytic end-feet by decreasing AQP4 mRNA. The activation of PKC with phorbol 12-myristate 13-acetate (PMA) reduces cerebral edema and AQP4 expression dose-dependently [114]. AQP4 localization in astrocytic end-feet relies on proteins such as α-syntrophin and dystrophin. In α-syntrophin knockout mice, the reduced expression of AQP4 results in less brain edema, highlighting the importance of α-syntrophin in the proper functioning of AQP4 [115]. CNS injury induces astrogliosis, marked by increased AQP4, GFAP, and V1aR expression. V1aR inhibition reduces brain edema and astrocyte swelling, suggesting its role in cytotoxic edema [106,116,117,118]. Oxidative stress further contributes by modifying AQP4 through processes like carbonylation, affecting its water transport efficiency and cellular homeostasis [119].

Brain injury-induced swelling causes hypoxia, and during early ischemia, anaerobic glycolysis and lactic acid production lead to acidosis that involves the exchange of protons for extracellular Na+ via Na+/H+ exchangers and Cl−/HCO_3_− antiporters [106]. Moreover, increased glutamate release from necrotic tissues is transported via the Na+ gradient [120,121]. To balance the influx of osmoles, glial cells absorb water primarily through AQP4. Hypoxia Inducible Factor-1α (HIF-1α), a mast nuclear transcription factor, is upregulated in response to the hypoxic conditions. Moreover, the accumulation of HIF-1α enhances NLRP3 inflammasome-mediated pyroptosis and activates microglia [122]. HIF-1α activates the transcription of various genes, including Vascular Endothelial Growth Factor (VEGF), which plays a role in angiogenesis and vascular permeability. The co-expression of VEGF and AQP4 in astrocytes is particularly critical in the formation and resolution of brain edema. Experimental studies have demonstrated that the overexpression of VEGF can lead to increased AQP4 expression, which in turn exacerbates edema formation in injured brain regions. It was also proven in laboratory setting that HIF-1alpha upregulates expression of both AQP4 and AQP9 [123]. 

Sirtuin 2 (SIRT2) belongs to the sirtuin family of NAD(+)-dependent protein deacetylases and serves as a critical modulator of AQP4. Sirt2 influences neuronal injury and neuroinflammation by regulating the expression of AQP4 through mechanisms that involve the deacetylation of specific transcription factors, leading to their activation or repression. The SIRT2 inhibitor AK-7 worsens traumatic brain injury (TBI) through a mechanism that likely includes enhanced acetylation and the nuclear movement of NF-κB p65, leading to an increased expression of NF-κB target genes such as AQP4, matrix metalloproteinase 9 (MMP-9), and pro-inflammatory cytokines [21,124].

In addition to changes in gene expression, post-translational modifications of AQP4 following TBI also influence its function. The phosphorylation of AQP4 can alter its cellular localization, affecting its water transport efficiency. The mislocalization of AQP4 has been linked to dysfunctional water transport and worsened edema [38,125]. The regulation of AQP expression in response to TBI is further complicated by the involvement of epigenetic modifications, such as DNA methylation and histone acetylation, which can alter gene expression without changing the DNA sequence [126,127]. These modifications can be triggered by environmental factors, including TBI, and influence long-term gene expression patterns.

TBI also influences AQP1 expression through molecular pathways involving PKA and PKC, activated by arginine vasopressin V1a receptors in response to elevated arginine vasopressin (AVP) levels. These pathways facilitate the phosphorylation of AQP1, promoting its rapid translocation to the cell membrane, thereby increasing water permeability and contributing to neuronal swelling. This regulatory mechanism highlights the potential of V1a receptors and AQP1 as therapeutic targets for managing brain edema following TBI [86].

Overall, the regulation of AQP expression in response to TBI is a multifaceted process involving immediate and delayed molecular responses. Understanding these mechanisms provides insights into the pathological processes following TBI and offers potential therapeutic targets to control brain edema and improve patient outcomes.

## 5. Diagnostic and Prognostic Implications

Aquaporins, particularly AQP4, have garnered significant interest not only for their roles in the pathophysiology of traumatic brain injury (TBI) but also as potential biomarkers for diagnosis, prognosis, and treatment monitoring. 

The unique properties and roles of aquaporins in water regulation make them attractive candidates as biomarkers in TBI. Given their critical involvement in the mechanisms underlying cerebral edema and BBB integrity, changes in AQP expression could potentially reflect injury severity and the risk of secondary complications.

Early diagnosis of TBI, particularly mild cases that may lack clear clinical manifestations, could be improved by detecting alterations in AQP levels in body fluids.

The degree of AQP alteration post-injury might predict the progression of cerebral edema and the overall prognosis, offering insights into the likely recovery trajectory and potential complications.

As therapeutic strategies targeting AQPs are developed, monitoring their levels could serve to assess the effectiveness of such treatments, adjusting them in real time to optimize outcomes.

Research into the detection of AQPs in the cerebrospinal fluid (CSF) and blood of TBI patients provides insights into their potential as biomarkers. Several studies have reported elevated AQP4 levels in the CSF of patients following TBI, correlating these levels with injury severity and outcomes (Table 1). For instance, a significant increase in AQP4 CSF levels has been observed in patients with severe TBI, associated with poor prognosis and higher rates of mortality. Similarly, changes in AQP4 levels in blood samples have been documented. Although less direct than CSF measurements due to the peripheral expression of AQP4, these studies still suggest a potential systemic response to brain injury that could be harnessed for diagnostic purposes. Moreover, research has begun to explore other aquaporin family members, such as AQP1 and AQP9, which are also altered in TBI and could provide additional or complementary diagnostic and prognostic information.

## 6. Emerging Technologies and Future Directions 

According to the guidelines of the Brain Trauma Foundation, the current goal in treating brain edema is to reduce intracranial pressure while maintaining adequate cerebral perfusion pressure (CPP). Available methods—hyperosmolar therapy, sedation, neuromuscular blockade, hypothermia, and decompressive craniectomy—act indirectly, are non-targeted, have a wide range of side effects, and can be implemented only in cases of established edema [138].

To date, no drug targeting the molecular basis of the edema formation process has been introduced, partly due to the dynamic nature of this process—AQP4 initially causes cytotoxic edema in the early phase but later is responsible for water removal during vasogenic edema [139]. However, several substances show promising effects on the pathophysiology of brain edema and could, if proven effective, provide useful tools for clinicians and minimize the need for symptomatic therapies (Table 2). Controlling astrocyte volume through the modulation of aquaporin function is key to minimizing the harmful effects of edema [140].

In preclinical studies, edema reduction has been achieved through the gene deletion of AQPs in laboratory rodents [141]. However, there is no direct inhibitor that effectively blocks the AQP4 channel to disable its physiological function. Substances that indirectly affect AQP channels and alter their functionality, thus effectively reducing edema, have been tested.

Bumetanide inhibits NKCC1 and reduces astrocyte swelling in vitro after fluid percussion injury (FPI) [142]. AER-271 blocks acute brain edema and improves early outcomes in a pediatric animal model of cardiac arrest due to asphyxia [143]. It improves prognosis and reduces edema in traumatic models complicated by brain edema due to overhydration and ischemic stroke caused by middle meningeal artery occlusion [85].

Aquaporumab inhibits the progression of neuromyelitis optica in in vivo studies but has not been tested in ischemic and traumatic models. Newer in vivo studies have shown that targeting the subcellular localization of AQP4, rather than directly targeting channel activity, significantly reduces AQP4 surface expression and associated edema, offering an alternative approach to anti-edema therapy [144].

### 6.1. Vasopressin V1a Receptors 

Since AVP receptors play a role in AQP-mediated water transportation within the kidney and inhibiting V1a receptors decreases the formation of brain edema following trauma, Rauen et al. hypothesized that V1a receptors might regulate cerebral AQPs. To investigate this, cerebral AQP1 and AQP4 messenger ribonucleic acid (mRNA) and AQP1 as well as AQP4 protein levels were quantified in wild-type and V1a receptor knockout (V1a −/−) mice before and 15 min, 1, 3, 6, 12, or 24 h after experimental TBI by controlled cortical impact. In non-traumatized mice, AQP1 and AQP4 expression in cortical neurons and astrocytes were measured, respectively. The established connection between AQP1 and AVP might stem from the existence of four phosphorylation sites on AQP1, which act as focal points for short-term regulation through protein kinase A (PKA) and protein kinase C (PKC). V1a receptors activate PKC, and in Xenopus oocytes, the water and ion permeability of AQP1 are enhanced through the PKC-dependent phosphorylation of threonine-157 and threonine-239.5. Furthermore, the phosphorylation of tyrosine-253 in the C-terminal domain appears to act as a pivotal control, governing the ion permeability of AQP1 through cyclic guanosine monophosphate. This mechanism is indirectly triggered by AVP via V1a receptors, inducing an elevation in intracellular calcium concentration. Hence, the reversible insertion of AQP1 into cell membranes induced by phosphorylation is likely dependent on AVP and V1a receptors, aligning well with the presented outcomes indicating the V1a receptor-mediated upregulation of AQP1 within 15 min post-experimental TBI. The prolonged elevation of AQP1 by V1a receptors in-volves various pathways, including c-Jun N-terminal kinase (JNK). Activation of JNK through AVP signaling leads to blood–brain barrier impairment and vasogenic brain edema. The presented study resulted in observations that V1a receptors control both short-term and long-term AQP1 expression and propose V1a receptors and AQP1 as promising therapeutic targets for managing cytotoxic brain edema following trauma. On the other hand, the absence of V1a receptors has no impact on AQP4 mRNA and AQP4 protein levels, thus preserving the beneficial AQP4-mediated removal of solutes, water, and macromolecules that could potentially worsen brain edema and post-trauma neurodegeneration [86]. 

### 6.2. Antisense Oligonucleotides

The study presented by Hekimoglu et al. aimed to disrupt the association of functional channel proteins, synthesized through AQP4 expression, with the cell membrane using antisense oligonucleotides (ASOs). ASOs bind to mRNA following Watson–Crick’s double-stranded principle, forming a steric hindrance that inhibits translation. The hybrid formed by ASOs and mRNA undergoes enzymatic degradation facilitated by RNAaseH activity, which operates through the RNAaseH mechanism. ASOs feature phosphodiester structures. Oligonucleotides with unmodified phosphodiester bonds degrade rapidly in biological fluids due to endo- and exonucleases. Various modifications have been devised to enhance in vivo stability and prevent degradation. ASOs may hinder the transcription of the binding site of AQP4 to the cell membrane during AQP4 mRNA synthesis. Consequently, this intervention could impede the formation of functional AQP4 combined with a membrane receptor without reducing AQP4 expression, thereby reducing channel function and improving edema. Treatment with ASOs resulted in reduced edema immediately after trauma at 0 h, yielding significant outcomes. However, no improvements were observed four hours post-treatment. It is plausible that intervening early, prior to the onset of cytotoxic edema within hours of trauma, could yield more promising results. Hence, adjusting the dosage of the antisense or altering the timing of administration might warrant consideration [145].

### 6.3. Minocycline

Minocycline is a broad-spectrum semi-synthetic tetracycline antibiotic that presents with anti-inflammatory, anti-apoptotic, vascular protection and neuroprotective properties in TBI models. Lu et al. explored the mechanism of minocycline treatment for TBI, particularly focusing on the interaction between minocycline and AQP4 during TBI therapy. There was a notable increase in brain water content in the TBI group compared to the control group. The assessed AQP4 protein expression in the cortex was notably suppressed by minocycline treatment. Under relatively normal conditions, AQP4 exhibited punctate reactivity around astrocytic end-feet. Post-TBI, astrocytes surrounding the lesion displayed swollen cell bodies, accompanied by the translocation of AQP4 from astrocytic processes to cell bodies. Remarkably, minocycline treatment reversed this translocation of AQP4 in astrocyte foot processes. Furthermore, significantly heightened AQP4 intensity and the diminished co-localization of GFAP and AQP4 in the TBI group were observed compared to the control group, signifying a loss of AQP4 in astrocytic end-feet post-TBI. Minocycline treatment decreased the fluorescent intensity of AQP4 and reinstated the co-localization of GFAP and AQP4. Collectively, these findings suggest that minocycline administration suppresses the expression of AQP4 in astrocyte foot processes following TBI. Distinct function of AQP4 in preserving BBB integrity related to TBI was also examined by employing both an AQP4 agonist (cyanamide, CYA) and TGN-020—an AQP4 inhibitor. The impact of three medications on AQP4 expression was assessed through Western blot analysis. AQP4 expression was notably elevated in the co-treatment group of minocycline and cyanamide compared to the minocycline-alone group. Conversely, the cyanamide-alone group exhibited a significant increase in AQP4 expression compared to the minocycline-alone group, while no significant difference in AQP4 expression was observed between the TBI + cyanamide-alone group and the TBI-alone group. In contrast, the administration of TGN-020 following TBI markedly reduced the expression of AQP4. The investigation of BBB integrity was performed by assessing the expression of tight junction proteins and MMP-9. The result showed that both minocycline and TGN-020 exhibited a significant upregulation of tight junction proteins (ZO-1, Occludin, and Claudin-5). The researchers demonstrated that minocycline effectively reduces the increased AQP4 levels induced by TBI, thereby protecting BBB integrity and modulating astrocyte characteristics following TBI [146].

### 6.4. Acetazolamide

Acetazolamide, an inhibitor of sulfonamide carbonic anhydrase (CA), impedes AQP4 water conductance through liposomes and hinders AQP4 upregulation following TBI. The prevention of AQP4 redistribution in a cultured TBI model in human astrocytes using acetazolamide was investigated by Glober et al. Treatment with acetazolamide prevented the punctate aggregation of AQP4. These findings indicate that acetazolamide modulates AQP4 distribution and potentially its function in humans. Given that acetazolamide prevents the rearrangement of AQP4 after TBI, the treatment effect on cytotoxic edema in closed-cortical injury model was investigated at 24 h post-TBI. Vasogenic edema was evaluated by measuring brain water content. Cytotoxic edema was evaluated using Nissl staining of frozen tissue sections. Study findings revealed a significant increase in the size of neuronal cell bodies within the cortex and CA1 region of the hippocampus of mice subjected to TBI compared to the control group, indicative of cytotoxic edema. In contrast, mice treated with acetazolamide exhibited cell bodies comparable to those from the control group. Overall, these results demonstrate that acetazolamide mitigates cytotoxic edema after TBI [147].

### 6.5. miR-211-5p

The dysregulated expression of matrix metalloproteinase 9 (MMP9) and AQP4 correlates with the progression of TBI. Wang et al. investigated the correlation between miR-211-5p and the MMP9/AQP4 axis in both TBI patients and astrocyte cells. Luciferase activity assays and gene expression analyses were conducted to elucidate the regulatory mechanism of miR-211-5p on MMP9/AQP4 in human astrocyte cells. The presented results showed that the mRNA levels of miR-211-5p were notably reduced in the CSF of TBI patients, exhibiting a positive correlation with the expression of both MMP9 and AQP4. In SVG P12 cells, miR-211-5p was found to directly target MMP9. Overexpression of miR-211-5p led to a decrease in MMP9 expression, while the inhibition of miR-211-5p through inhibitors resulted in increased expression levels of both MMP9 and AQP4. The consequence of the miR-211-5p inhibitory effect on the MMP9/AQP4 axis in human astrocyte cells leads to a promising therapeutic strategy for TBI treatment [148]. 

### 6.6. Trifluoperazine

Trifluoperazine (TFP) is a pharmaceutical antipsychotic agent. Studies have revealed that TFP mitigates central nervous system edema by impacting the subcellular localization of AQP4 in astrocytes [149]. However, the precise mechanism of action of TFP in TBI remains incompletely understood. In the study conducted by Xing et al., immunofluorescence co-localization analysis revealed a significant increase in the area and intensity covered by AQP4 on the surface of brain cells, specifically astrocyte end-feet, following TBI. Conversely, TFP treatment reversed these changes, indicating its inhibitory effect on AQP4 accumulation at the brain cell surface. The tunnel fluorescence intensity and fluorescence area were lower in the TBI + TFP group compared to the TBI group. Furthermore, the TBI + TFP group exhibited reduced brain edema, a brain defect area, and a modified neurological severity score (mNSS) compared to the TBI group [150]. Comparable results were obtained by Sylvain et al. The impact of TFP treatment on AQP4 gene expression in the brain was assessed. AQP4 mRNA levels in the lesion were quantified and the contralateral cortex of stroke animals was compared to the cortex from sham animals, with TFP administered either 30 min before stroke or 1 h after stroke. It was determined that TFP treatment 1 h post-stroke significantly reduces AQP4 mRNA expression levels in the stroke lesion compared to the lesion in non-treated injured animals. Interestingly, the contralateral cortex in non-treated injured animals and pre-treated stroke animals exhibited significantly higher AQP4 mRNA levels compared to the contralateral cortex from stroke animals treated with TFP at 1 h post-stroke. This study represents the evidence that TFP effectively diminishes brain water content during the acute phase of stroke in mice utilizing the photothrombotic model. These neuro-protective benefits were observed only when TFP was administered after, rather than before, the onset of stroke and during the acute phase of cerebral edema. TFP’s capacity to reduce brain swelling and edema may be attributed to its inhibitory actions on AQP4 expression at both gene and protein levels in post-stroke animals [151]. 

### 6.7. Adenine Dinucleotide Phosphate Oxidase 2

The involvement of nicotinamide adenine dinucleotide phosphate oxidase 2 (NADPH oxidase 2; NOX2) and AQP4 in edema formation and cognitive function following TBI in NOX2−/− and AQP4−/− mice was investigated by He et al. utilizing the Morris water maze test, step-down test, novel object recognition test, and Western blotting. NOX2 knockout in mice led to reduced AQP4 levels and decreased edema in the hippocampus and cortex post-TBI. Furthermore, the inhibition of AQP4 by 2-(nicotinamide)-1,3,4-thiadiazole (TGN-020) or genetic deletion of AQP4 attenuated neurological deficits without altering reactive oxygen species levels post-TBI in mice. Researchers concluded that NOX2 inhibition could enhance cognitive function by modulating reactive oxygen species levels, subsequently impacting AQP4 expression and brain edema following TBI in mice [152].

### 6.8. Hypertonic Saline

The reduction in AQP4 expression and pro-inflammatory cytokines like tumor necrosis factor (TNF-α) and interleukin (IL-1β) has been associated with the development of edema. A study conducted by Yin et al. investigated the impact of 3% HS on brain edema in a rat model of traumatic brain injury. The hypertonic saline group received injections of 3% NaCl. AQP4, TNF-α, IL-1β, and caspase-3 levels were assessed using Western blotting, immunohistochemistry, enzyme-linked immunosorbent assay, and quantitative real-time PCR. Brain water content was also evaluated. Terminal deoxynucleotidyl transferase dUTP nick-end labeling was employed to detect apoptotic cells in brain tissue. TBI induced increases in caspase-3 mRNA expression and the number of apoptotic cells; however, treatment with 3% HS suppressed apoptosis compared to the TBI group. IL-1β spurred AQP4 expression in both the microglia and astrocytes, while TNF-α emerged as a pivotal mediator of brain edema. Treatment with 3% HS led to a decrease in brain water content compared to the TBI group. Moreover, decreases in AQP4, TNF-α, and IL-1β mRNA and protein levels were observed [153]. 

### 6.9. Monocyte Locomotion Inhibitor Factor

Monocyte locomotion inhibitor factor (MLIF) is a small molecular pentapeptide that has shown promising results in protecting against cerebral ischemia [154]. It led to the exploration of the protective effects of MLIF on TBI and the investigation of its underlying mechanism of action by Li X. et al. In animal experiments, the administration of MLIF post-TBI resulted in reduced brain water content and improved brain edema, indicating a degree of protection against TBI. Using network pharmacology methodologies, potential targets of MLIF in the context of TBI were screened, identifying ten signaling pathways closely associated with TBI. Through molecular docking techniques, AQP4 was pinpointed as one of the top ten central genes discovered in this study. Findings revealed that MLIF possesses anti-apoptotic properties and suppresses the expression of AQP4 protein, thereby playing a protective role in traumatic brain injury [155]. 

### 6.10. Lentivirus-Mediated AQP4 Gene Silencing

In the study conducted by Li B et al., AQP4 expression in the brain was suppressed by injecting an AQP4 shRNA-lentiviral vector. Western blot and qRT-PCR were employed to assess the expression of relevant genes. Neuronal apoptosis was evaluated using a TUNEL assay. Treatment with AQP4 shRNA reduced AQP4 expression in the brains of TBI rats. AQP4 shRNA mitigated TBI-induced brain edema and neurological deficits in rats. Neuronal apoptosis and astrocyte activation in TBI rats were diminished by AQP4 silencing. This study demonstrated that silencing AQP4 using AQP4 shRNA in the TBI rat model decreased the expression of AQP4 and GFAP; attenuated brain edema, neuro-logical deficits, and neuronal apoptosis; and inhibited astrocyte activation [156].

### 6.11. Omega-3 Polyunsaturated Fatty Acids

Omega-3 polyunsaturated fatty acids (Omega-3 PUFAs) play a role in clearing amyloid-ß via the glymphatic system, and this process relies on AQP4. In the study conducted by Zhang et al., mice were pretreated with Omega-3 PUFAs-rich fish oil and subjected to TBI. Neurological functions were evaluated using the modified neurological severity score (mNSS) system and Rota-rod test. Levels of Aß42 and radioisotope clearance were assessed to determine glymphatic system function. AQP4 protein and mRNA expressions, as well as polarity, were examined in TBI mice treated with fish oil or controls. The integrity of the blood–brain barrier was assessed through Evans blue extravasation and the measurement of tight junction proteins (ZO-1 and Occludin) levels. TBI surgery induced significant neurological functional impairment, which was attenuated by Omega-3 PUFAs, as demonstrated by reduced mNSS and improved performance in the Rota-rod test. Omega-3 PUFAs also improved glymphatic clearance post-TBI, reduced Aß42 accumulation, and partially restored the clearance of both 3H-mannitol and 14C-Inulin. Furthermore, Omega-3 PUFAs suppressed AQP4 expression and partially prevented the loss of AQP4 polarity in TBI mice. As was stated in conclusion: Omega-3 PUFAs protected mice from TBI-induced blood–brain barrier disruption [157]. 

### 6.12. Angiotensin II Type 1 Receptor

The study conducted by Yang et al. aimed to investigate the impact of AT1 receptor deficiency on the BBB in TBI mice, as well as its effect on Aβ levels and glial lymphatic circulation. A TBI model was established using AT1 receptor knockout mice (AT1-KO) and C57BL/6 mice (wild-type, WT). BBB integrity was assessed using Evans blue extravasation. Immunofluorescence was utilized to evaluate the expression of the astrocytic water channel AQP4 and astrocyte activation. The levels of amyloid precursor protein (APP), the junction proteins zonula occludens protein-1 (ZO-1), and occludin in mouse brains were measured via Western blot (WB). Aβ levels were determined using enzyme-linked immunosorbent assay (ELISA). Deficiency in the AT1 receptor preserved BBB integrity and mitigated the decrease in occludin and ZO-1 expression induced by TBI in mouse brains. AT1-KO mice exhibited less pronounced increases in APP expression, Aβ 1–42, and Aβ 1–40 levels compared to WT mice following TBI. Furthermore, AT1 receptor deficiency significantly inhibited AQP4 depolarization post-TBI. It was observed that AT1 receptor deficiency attenuated TBI-induced BBB impairments by preserving tight junction proteins and inhibiting AQP4 polarization, thereby enhancing the glymphatic system’s function and improving interstitial Aβ clearance in the brains of TBI mice [158].

### 6.13. Melatonin Receptors

The research from 2018 conducted in Iran showed that the reduction of cerebral edema by estrogen may be linked to the function of melatonin receptors (MT1, MT2, and MT3). Moreover, melatonin receptor activity (MT1 and MT2) likely contributes to the decrease in BBB permeability induced by estrogen and the interaction between melatonin receptors (MT2 and MT3) and estrogen may affect intracranial pressure (ICP). The AQP4 levels increased in the melatonin receptor antagonist vehicle+ estrogen group compared to the trauma group. The AQP4 concentration in the melatonin antagonist vehicle+ estrogen did not differ from that in the prazosin + estrogen, 4-phenyl-2-propionamido-tetralin+ estro-gen, and luzindole+ estrogen groups. It was concluded that melatonin receptors do not participate in the influence of estrogen on the expression of AQP4 [159].

Both stem cells and miRNAs strongly regulate AQP4 expression, providing new tools for influencing water balance following brain injuries [160]. 

### 6.14. Other Targets

Molecules like agrin and laminin are vital for BBB integrity and regulate the polarization of AQP4 within glial cells. A unique aspect of AQP4 at the perivascular end-feet is its display of polarized and densely packed formations (termed orthogonal arrays) of particles. When primary mouse astrocytes were cultured with the neuronal agrin paralog A4B8, there was an increase in AQP4 membrane clustering in orthogonal arrays of particles, along with elevated levels of the M23 splice variant. A similar effect was observed in primary rat astrocytes in the presence of laminin. In mice lacking endothelial agrin, surface levels of AQP4 were downregulated. Additionally, the genetic deletion of dystrophin or α-syntrophin resulted in the loss of AQP4 polarization in mice, providing protection against edema formation compared to the control group. These findings indicated the importance of membrane tethering and polarization within astrocytes for AQP4 function. Following neuronal damage, reactive astrocytes migrate to lesion sites in an AQP4-dependent manner and form glial scars to isolate dying neurons, limiting tissue damage spread. Astrocytes from wild-type mice exhibited a three-fold higher migration rate and 50% faster wound-healing rate compared to those from AQP4−/−, mice leading to a conclusion that AQP4 is crucial for astrocyte plasticity [161].

**Table 2:** The table summarizes various emerging technologies for treating brain edema, highlighting current methods, promising drugs, and approaches, along with references to supporting studies.

Technology/Approach	Description	Reference
Gene deletion of AQPs	Reduces edema in preclinical studies but lacks direct AQP4 inhibitor	[141]
Bumetanide	Inhibits NKCC1, reduces astrocyte swelling after fluid percussion injury	[142]
AER-271	Blocks acute brain edema, improves early outcomes in pediatric cardiac arrest models	[143]
Aquaporumab	Inhibits neuromyelitis optica progression in vivo, not tested in ischemic/traumatic models	[144]
Vasopressin V1a receptors	Regulates AQPs in the brain, potentially controlling cerebral edema through PKC activation and JNK pathways	[86]
Antisense oligonucleotides	Bind to mRINA, prevent AQP4 functional protein formation, reduce edema if administered early	[145]
Minocycline	Suppresses AQP4 expression post-TBI, protects BBB integrity, modulates astrocyte characteristics	[146]
Acetazolamide	Inhibits CA, prevents AQP4 redistribution post-TBI, mitigates cytotoxic edema	[147]
miR-211-5p	Regulates MMP9/AQP4 axis, therapeutic potential for TBI treatment by targeting this pathway	[148]
Trifluoperazine	Reduces AQP4 accumulation, mitigates brain edema, reduces neurological severity post-TBI	[149,150,151]
Adenine dinucleotide phosphate oxidase 2	NOX2 inhibition reduces AQP4 levels, enhances cognitive function, reduces brain edema post-TBI	[152]
Hypertonic saline	Reduces AQP4, TNFa, IL-1B levels, and brain water contact, suppresses apoptosis post-TBI	[153]
Monocyte locomotion inhibitor factor	Reduces brain water content, suppresses AQP4 suppression, provides protection against TBI	[154,155]
Lentivirus-mediated AQP4 gene silencing	Reduces AQP4 expression, mitigates brain edema, reduces neurological deficits and neuronal apoptosis post-TBI	[156]
Omega-3 polyunsaturated fatty acids	Improve glymphatic clearance, reduce AB42 accumulation, protect BBB integrity post-TBI	[162]
Angiotensin II type 1 receptor	Deficiency preserves BBB integrity, reduces AQP4 polarization, improves glymphatic function, and AB clearance post-TBI	[158]
Melatonin receptors	Interaction with estrogen may reduce BBB permeability, influence AQP4 expression	[160]
Arginin and laminin	Regulate BBB integrity and AQP4 polarization, important for astrocyte migration and plasticity	[161]

Future research should focus on developing specific inhibitors and modulators of AQP channels, particularly AQP4, to directly intervene in the edema formation process. Additionally, the exploration of gene silencing techniques, such as the use of antisense oligonucleotides, and the modulation of aquaporin expression through pharmacological agents or dietary supplements like Omega-3 PUFAs could provide new therapeutic avenues. Moreover, the study of receptor interactions, such as the AT1 receptor and melatonin receptors, offers promising strategies to control AQP function and address brain swelling and BBB integrity post-TBI.

## 7. Conclusions

The exploration of aquaporins, particularly AQP4, in the context of traumatic brain injury (TBI) has unveiled a critical avenue for potentially mitigating the severe consequences associated with this condition. The modulation of AQPs, especially AQP4, holds substantial promise for improving TBI outcomes by directly addressing one of its most challenging complications: cerebral edema.

Modulating AQP4 could control the flow of water into and out of brain cells, potentially reducing the severity of edema and thereby decreasing intracranial pressure—one of the primary causes of mortality in severe TBI cases. By mitigating edema and the associated stress on brain tissues, AQP modulation could enhance the viability of neurons and other brain cells post-injury, leading to improved recovery and functional outcomes. Targeting AQPs allows for a more tailored therapeutic approach based on individual variations in AQP expression and injury specifics, embodying the principles of precision medicine in neurology.

Despite the promising potential of AQP modulation in TBI treatment, several research and development steps must be undertaken before these strategies can be translated into clinical practice. Continued research is needed to elucidate the detailed mechanisms by which AQPs influence cerebral edema and other TBI pathologies. This includes understanding the differential roles of various AQPs in different types and severities of brain injury. There is a critical need for the development and refinement of AQP modulators, both inhibitors and enhancers, that are selective and effective under physiological conditions. These agents must be capable of crossing the BBB and reaching their targets without significant off-target effects. Effective delivery mechanisms specifically tailored for brain tissue are essential. Rigorous preclinical testing in animal models followed by well-designed clinical trials in human subjects are essential to validate the efficacy and safety of AQP modulators. These studies will also help refine dosing regimens, administration methods, and therapeutic windows. Collaboration with regulatory bodies to develop guidelines and obtain approvals for new treatments is crucial. This includes establishing protocols for clinical use and monitoring treatment outcomes in real-world settings.

## 8. Final Thoughts

Modulating AQPs represents a frontier in the treatment of TBI that aligns with the evolving understanding of molecular and cellular neurobiology. As research progresses, the insights gained will not only pave the way for innovative treatments but also enhance our broader understanding of brain health and disease. The journey from laboratory benches to hospital bedsides, while challenging, is filled with the promise of significant advancements in the care and recovery of TBI patients.

## Figures and Tables

**Table 1:** The table includes a list of studies investigating various aquaporins in the context of traumatic brain injury, providing insights into their roles, sample types, and the nature of the studies conducted.

Subject	Key Findings	AQP Type	Sample Type	Study Type	Year
“Cellular distribution of brain aquaporins and their contribution to cerebrospinal fluid homeostasis” [128]	Explored changes in aquaporin expression post-TBI in rats	AQP4	Brain tissue, CSF	Experimental	2022
“Dynamic regulation of aquaporin-4 water channels in neurological disorders” [107]	Analyzed AQP4 expression changes in CSF of severe head injury patients	AQP4	CSF	Clinical	2015
“Immunohistochemical evaluation of aquaporin-4 and its correlation” [129]	Examined AQP4 expression and localization changes in TBI patients	AQP4	Brain tissue	Clinical	2018
“Aquaporin-4 distribution in control and stressed astrocytes” [130]	Found elevated AQP4 levels in CSF after TBI	AQP4	CSF	Experimental	2013
“Neuronal damage and functional deficits are ameliorated by inhibition of aquaporin” [131]	Linked changes in AQP expression post-TBI to functional outcomes	AQP4	Brain tissue	Experimental	2012
“Effect of decompressive craniectomy on aquaporin-4 expression” [132]	Investigated AQP4 expression changes following TBI	AQP4	Brain tissue	Experimental	2011
“Aquaporin 4 expression and ultrastructure of the blood–brain barrier” [133]	Studied the dynamic change of AQP4 expression after cerebral contusion injury	AQP4	Brain tissue	Experimental	2013
“Protective effects of aquaporin-4 deficiency on neurological outcomes” [134]	Studied the effects of AQP4 deficiency on TBI outcomes	AQP4	Brain tissue	Experimental	2021
“Fluid-percussion brain injury induces changes in aquaporin channel expression” [135]	Examined the changes in AQP expression levels in a rat model of TBI	Multiple AQPs	Brain tissue	Experimental	2011
“Regulation of aquaporin-4 in a traumatic brain injury model in rats” [80]	Explored the regulation of AQP4 expression in TBI and its clinical implications	AQP4	Brain tissue	Experimental	2003
“Emerging roles for dynamic aquaporin-4 subcellular relocalization” [19]	Discussed the dynamic relocalization of AQP4 in CNS water homeostasis post-TBI	AQP4	Brain tissue, CSF	Review	2022
“Expression of aquaporin-4 and pathological characteristics of brain injury” [136]	Explored the relationship between AQP4 expression and brain injury pathology	AQP4	Brain tissue, CSF	Experimental	2015
“Aquaporin and brain diseases” [137]	Examined the impact of traumatic brain injury on aquaporin expression levels	Multiple AQPs	Brain tissue, CSF	Review	2014
